# Deaf individuals who work with computers present a high level of visual attention

**DOI:** 10.1590/S1980-57642011DN05020011

**Published:** 2011

**Authors:** Paula Vieira Ribeiro, Valdenilson Ribeiro Ribas, Renata de Melo Guerra Ribas, Teresinha de Jesus Oliveira Guimarães de Melo, Carlos Antonio de Sá Marinho, Kátia Karina do Monte Silva, Elizabete Elias de Albuquerque, Valéria Ribeiro Ribas, Renata Mirelly Silva de Lima, Tuthcha Sandrelle Botelho Tavares Santos

**Affiliations:** 1 Masters in Human Resource Management and Organizational Behavior; 2 Doctor in Neuropsychiatry; 3 Expert Sanitary Surveillance; 4 Masters in Human Resource Management and Organizational Behavior; 5 Expert in School Education and Educational Planning; 6 Doctor in Neuroscience by Georg-August Universitat Gottingen; 7 Masters in Human Resource Management and Organizational Behavior; 8 Masters in Neuropsychiatry; 9 Graduate Student in Linguistics; 10 Masters Student in Social-Organizational Psychology

**Keywords:** deaf, attention, computers

## Abstract

Some studies in the literature indicate that deaf individuals seem to develop a higher level of attention and concentration during the process of constructing of different ways of communicating.

**Objective:**

The aim of this study was to evaluate the level of attention in individuals deaf from birth that worked with computers.

**Methods:**

A total of 161 individuals in the 18-25 age group were assessed. Of these, 40 were congenitally deaf individuals that worked with computers, 42 were deaf individuals that did not work, did not know how to use nor used computers (Control 1), 39 individuals with normal hearing that did not work, did not know how to use computers nor used them (Control 2), and 40 individuals with normal hearing that worked with computers (Control 3).

**Results:**

The group of subjects deaf from birth that worked with computers (IDWC) presented a higher level of focused attention, sustained attention, mental manipulation capacity and resistance to interference compared to the control groups.

**Conclusion:**

This study highlights the relevance sensory to cognitive processing.

## Abstract (pt)

Há trabalhos na literatura sinalizando que surdos parecem desenvolver maior nível de atenção e concentração durante a sucessão da construção das diversas formas de comunicação.

**Objetivo:**

Avaliar o nível de atenção em pessoas surdas desde o nascimento que trabalham com os computadores.

**Métodos:**

Foram avaliadas 161 pessoas, na faixa etária, entre 18 e 25 anos, sendo 40 pessoas surdas desde o nascimento que trabalham com computadores, 42 pessoas surdas que não trabalham, não sabem e nem utilizam computadores (Controle 1), 39 pessoas ouvintes que não trabalham, não sabem e nem utilizam computadores (Controle 2) e 40 pessoas ouvintes que trabalham com computadores (Controle 3).

**Resultados:**

As pessoas surdas desde o nascimento que trabalham com computadores PSNC apresentaram maior nível de foco de atenção, de manutenção do foco, capacidade de manipulação mental e resistência à interferência, quando comparadas aos controles.

**Conclusão:**

O estudo mostra a relevância dos sistemas sensoriais para o processamento cognitivo.

## Introduction

The organization of thought depends directly on the competence that an individual has in relation to their language, which acts as a type of a world and tool organizer to create and understand it. Thus, societies were organized, making possible the construction of what we understand as civilization, centered on the relationship among citizens and regulated by rules of civil law.^1^

With regard to civilization, its maintenance depends not only on communication, but also on the survival of each citizen, who in order to provide for their subsistence, must engage in some socioeconomic activity.

In this context, deaf individuals face a great difficulty because they are stigmatized as poor sufferers, and still rely on scientists who are divided over dichotomous concepts in a struggle that stymies the advancement of research, where speech therapists defend oral production, while educators defend sign language.^2^

Amid this impasse, there is another question that hampers further progress the deaf in Brazil regarding the process of social inclusion, namely, difficulty gaining access to specialized speech treatment due to high costs, and to schemes with Brazilian Sign Language (LIBRAS) owing to the low number of qualified professionals in urban centers.^3^

Hearing impairment can limit individual development, since hearing is essential for the acquisition of the oral language. Initially, deafness interferes in the relationship between mother and child and creates gaps in the psychological processes of integration of experiences that will affect, to a greater or lesser degree, the normal capacity of individual development. Although there are no definitive general statistics, the incidence of deafness cases is high. The School Census/2004 revealed 62,325 deaf children studying at schools nationwide. These figures highlighted the need of preparing educators to adequately cater to this population.^4^

Independently of deaf communication, some studies in the literature indicate that the deaf seem to develop a higher level of attention and concentration during the process of constructing different ways of communicating such as the process of lip-reading that engages the mental activity of the subject to rebuild the messages.^5^

Since the 50s, several theories of attention have been put forward, but the authors Mateer & Mapou (1996) devised a model that integrates all the previously proposed theories. Also, the form and the writing of their explanations are more comprehensible for the reader. These authors proposed a model that does not detach attention from memory, and yet divides it into two core factors, denominated deployment and encoding.^6,7^

### Deployment

Denotes the ability to channel attention to specific stimuli and then maintain attention on this stimuli. The deployment factor is observed and divided into three aspects: wakefulness level, focused and sustained attention.^8^

Wakefulness level, or surveillance, is related to the phase in which the individual is awake. Thus, it is observed in the wakefulness level whether the individual is awake, asleep, torpid or comatose. Focused attention on the other hand, is related to the capacity of an individual to select a stimulus from among others, that is, to select a stimulus of interest. Focused attention is assessed together with sustained attention and not separately. When an individual selects a stimulus, they intend to focus on that stimulus for a certain period. Sustained attention is the capacity to maintain focus during the time of interest. These are the abilities of deployment, namely, surveillance, focused and sustained attention.^9^

Focused attention is usually evaluated in conjunction with sustained attention, using tests that require the subject to choose a stimulus and keep tracking it. To this end, cancellation tests are used. One simple test used to evaluate focus is the digit symbol test, which consists of four lines, each containing 25 gaps to be filled, giving a total of 100 gaps. Each gap is associated to a randomly distributed number (1-9). Besides these lines, another is provided, with pairs of numbers (1-9) and symbols. The examined subject must fill in the gaps of the four lines according to these presented pairs. The 7 first gaps served as a trial exercise. The subject is asked to fill in all the other gaps, as fast as possible, within an interval of 90 seconds. In other words, the digit symbol requires the correct corresponding numbers, from 1 to 9, and their respective symbols, for 1 minute and 30 seconds. The score on the test is equal to the number of correct answers over this time interval. In adults, this test evaluates psychomotor performance and focused attention, being virtually unaffected by variables such as intellectual capacity, memory or learning. The interference of age is often evident after 60 years old. The test-retest reliability is high, with correlation coefficients ranging from 0.82 to 0.88.^10^

Sustained attention is typically evaluated using more elaborate tests, with a higher level of difficulty, such as the d2 test. The d2 is a test where the individual must mark all instances of letter d followed by two dashes, on a paper sheet containing several lines, letters and dashes. The dashes may be above or below the letter d or one above and another below the letter. What it is important is that the individual marks only the letter d followed by two dashes, and not any other letters like p, for example. This requires the ability to maintain the focus of attention to a greater degree than the trail making task. In addition, subjects must overcome the fatigue of searching for this target stimulus.^6^

### Attention capacity

Denotes the ability of the subject to store information in memory in a short period, and perform mental manipulation of the information. This factor is subdivided into three aspects: attention span, mental manipulation of information, and resistance to interference. Attention span relates to the quantity of information that the individual can temporarily store in memory. This test is also used as a span of verbal memory, short-term memory, because in this sector, the distinction between attention and memory begins to blur. Therefore, it is a measure of the individual’s capacity to store information for a short period. Subjects are assessed using tests with a span of digits, which start with a sequence of 3 digits and increase progressively, where subjects are also asked to repeat this sequence. On the last correctly repeated sequence, the quantity of the repeated digits is considered the span of the subject. Thus, if a subject repeats a sequence of up to 6 digits, they have a span of 6.^11^

This is an indirect form of measuring the quantity of information which the subject is able to retain. This is also a verbal span, for there is a span evaluation by visual memory, which is a similar task to using the Corsi block comprising a board with several squares. Behind these squares there are painted numbers, but only the examiner can see these numbers while the assessed subject cannot. The examiner points to a sequence of squares, following the numbers that are behind, where the evaluated subject does not see these numbers. Subjects must only repeat the sequence of touches of the examiner. Usually, this begins with three touches that the subject is able to reproduce correctly. This is the visual span (verbal).^8^

### Mental manipulation of information capacity

This means that the individual stored information for a short time and now needs to manipulate this information. For example, the use of span of digits in inverse order, which is similar to the digit span, but, instead of repeating the sequence of numbers that the examiner reads aloud, the patient must repeat the sequence in inverse order. Thus, if the examiner says, for example, 8 - 9 - 1, the patient must repeat 1 - 9 - 8, and so forth.^11^

The sequences of digits continue to increase in length and the patient repeats these digits in inverse order. Besides the maintenance of the information in memory, the subject must also mentally manipulate the information. Subjects must memorize the sequence, invert the order and then reproduce verbally aloud in order to be rated by the examiner as correct or otherwise. There are other tasks that enable the verification of the mental manipulation of information. Another example of this situation is a test called PASAT, which verifies the capacity of mental manipulation of information, besides the capacity of resisting interference.^6^

Resistance to interference is the third aspect that must be evaluated by the Paced Auditory Serial Addition Test (PASAT). In this test, the individual must add a sequence of numbers given to them. The examiner then says the first number, the second, and from this second number the evaluated subject must add this last number to the previous one. For example, if 9, 8=17 is given, when the next number is said the subject must add this number to the previous one (8 in this case) and not to the result of the previous sum (17 in this case). Thus, the subject must be able to maintain the last number of the information and discard the number produced, i.e. the previous sum. This test thus requires resistance to interference capacity.^12^

This background, and occupational relationship of these authors with deaf individuals that work with computers, led to the hypothesis that the deaf may have a higher level of visual attention than individuals with normal hearing. Thus, the aim of the present study was to investigate the attention level in deaf individuals that work with computers, and was carried out at the Department of Post-graduation in Human Resources of the Miguel Torga Institute in Portugal, in partnership with researchers of the Post-graduation course in Neuropsychiatry and Behavioral Sciences of the Federal University of Pernambuco in Brazil.

## Methods

### Subjects

A total of 161 individuals in the 18-25 age group were evaluated. The sample comprised 40 individuals deaf from birth who worked with computers (IDWC), 42 deaf individuals that did not work, did not know how to use nor used computers (Control 1), 39 individuals with normal hearing that did not work, did not know how to use nor used computers (Control 2) and 40 individuals with normal hearing that worked with computers (Control 3). The subjects were submitted to attention evaluations in a quiet room under standard conditions, in a building with ceiling fans, at a temperature of 29º±2ºC. Only male subjects were included, with female subjects excluded due to the difficulty finding deaf women working with computers.

### Evaluation

The instruments used for data collection were: Informed Consent (IC); identification test; focus of attention test - digit symbol; maintenance of focus test - d2; span of visual attention test - span of digits in direct order (adapted); mental manipulation test - span of digits in inverse order (adapted) and resistance to interference test (adapted) - paced auditory serial addition test (PASAT) adapted to paced visual serial addition test (PVSAT).

The data collection was carried at 7 a.m. before work, in order to avoid interference of tiredness. The project was approved by the Ethics Committee in Research of the Restoration Hospital in Recife/PE.

This evaluation was performed with the digit symbol, d2, span of digits in direct and inverse order and PVSAT instruments.

For evaluation of Attention Distribution, the digit symbol and d2 tests were applied.

For Attention Capacity, span of digits tests (digit span in forward and inverse) and the Paced Auditory Serial Addition Test (PASAT), were applied. The three tests were adapted to evaluations by sight. All numbers in the three tests were displayed on a projector. The PASAT was adapted to the Paced Visual Serial Addition Test (PVSAT).

**PVSAT** – This test verifies the capacity of resisting to interference in attention of the assessed subject. It entails the individual adding a sequence of numbers displayed on a projector i.e. whereas the PASAT is orally presented, the PVSAT is visually displayed.

### Statistics treatment

**Statistics program** – The program used to calculate the statistics was the SIGMA STAT for Windows - Version 2.0 of the Jandel Corporation.

**Data analysis** – Data were analyzed by the Analysis of Variance test (ANOVA), with p<0.05, expressed as mean± SEM (X±SEM).

## Results

**Evaluation of focused attention - Digit Symbol (WAIS III)**: Individuals deaf from birth that worked with computers (IDWC) presented a higher level of focused attention (49.40±1.07) compared to deaf that did not work, did not know how to use nor used computers (Control 1)(39.95±0.79) and to individuals with normal hearing that did not work, did not know how to use nor used computers (Control 2)(39.03±1.42), but no statistically significant difference was found compared to individuals with normal hearing that worked with computers (Control 3) (48.6±2.15) (Table 1).

**Table 1:** Evaluation of attention level in deaf individuals.

Subjects	Focused attention Mean±SEM	Sustained attention Mean±SEM	Mental manipulation Mean±SEM	Resistance to interference Mean±SEM
Control 1	39.95±0.79↓	478.02±10.91↓	4.17±0.15	6.50±0.58↓
Control 2	39.03±1.42↓	342.05±17.10↓	2.97±0.16↓	6.92±0.33↓
Control 3	48.6±2.15	428.40±9.28↓	4.88±0.17	8.68±0.19↓
IDWC	49.40±1.07*↑	567.80±10.01*↑	4.63±0.13*↑	9.43±0.56*↑

One hundred and sixty-one (161) subjects in the 18-25 year age group were evaluated. Of these, 40 were individuals deaf from birth who worked with computers (IDWC), 42 were deaf individuals that did not work, did not know how to use nor used computers (Control 1), 39 individuals with normal hearing that did not work, did not know how to use nor used computers (Control 2) and 40 individuals with normal hearing that worked with computers (Control 3). Statistics: ANOVA, p<0.05*.

**Evaluation of sustained attention - d2 test:** Individuals deaf from birth who worked with computers (IDWC) presented a higher level of maintenance of focus of attention (567.80±10.01) compared to deaf individuals that did not work, did not know how to use nor used computers (Control 1)(478.02±10.91), to individuals with normal hearing that did not work, did not know how to use nor used computers (Control 2)(342.05±17.10) and to individuals with normal hearing that worked with computers (Control 3)(428.40±9.28) (Table 1).

Evaluation of attention span - Span of digits in direct order: No statistically significant difference was detected among groups.

**Evaluation of mental manipulation capacity - Span of digits in inverse order:** Individuals deaf from birth that worked with computers (IDWC) presented a higher level of mental manipulation capacity (4.63±0.13) compared to individuals with normal hearing that did not work, did not know how to use nor used computers (Control 2) (2.97±0.16), but there was no statistical significant difference compared to deaf individuals who did not work, did not know how to use nor used computers, (Control 1) (4.17±0.15) and to individuals with normal hearing that worked with computers (Control 3) (4.88±0.17) (Table 1).

**Evaluation of resistance to interference capacity - paced auditory serial addition test (PASAT) adapted to paced visual serial addition test (PVSAT):** Individuals deaf from birth who worked with computers (IDWC) presented a higher level of resistance to interference capacity (9.43±0.56) compared to deaf individuals that did not work, did not know how to use nor used computers (Control 1) (6.50±0.58), to individuals with normal hearing that did not work, did not know how to use nor used computers (Control 2) (6.92±0.33) and to individuals with normal hearing that worked with computers (Control 3) (8.68±0.19) (Table 1).

## Discussion

This study showed that individuals deaf from birth that worked with computers (IDWC) presented a higher level of focused attention, sustained attention, mental manipulation and resistance to interference capacity compared to deaf individuals that did not work, did not know how to use nor used computers, to individuals with normal hearing that did not work, did not know how to use nor used computers, and to individuals with normal hearing that worked with computers.

These results corroborate the findings of Bavelier et al. (2000) and Menezes et al. (2009). However, although these results appear similar, it is necessary to report their methodological differences.^10,13^

Bavelier et al. (2000) studied a sample of individuals deaf from birth. Also, these authors did not employ psychological tests, but instead investigated, by applying magnetic resonance, to the selected area of movement linked to the visual field, involving the central and peripheral vision. Menezes et al. (2009) however, used exactly the same methodological tools as the present test, including the following tests: digit symbol, d2, Span of digits in direct and inverse order and PASAT. Their group however, did not study deaf subjects and instead focused on aspects related to auditory attention.^13^

Nevertheless, the results of this study present a curiosity that qualify observations made by Menezes et al. (2009), shown through the techniques of neuroimage, intensely used in the investigation of human brain functioning with emphasis on the use of positron emission tomography - PET and single photon emission computed tomography - SPECT. Both tests enable the construction of three-dimensional maps of brain activity from the gamma ray detection emitted by tracers marked with radioactive isotopes administered intravenously or inhaled.^14^

These authors established the possibility of alteration in the pattern of brain perfusion with the increased blood flow observed on exams of structural neuroimaging done after physical exercise ^15^ or during reading.^16^ In this context, it seems that the results of this study add further to the findings of Menezes et al. (2009), since the use of computers also allowed this type of brain functioning.

Their inferences draw on and ratify two earlier theories of the last century involving different approaches. The first is based on the imitation theory of Bandura (1969) apud Pellegrini et al., (1987). Bandura proposed that most social behavior is learned by the observation and imitation of the behavior of others, mainly father and mother.^17^

The second approach, although entailing another theoretical approach - psychoanalytical - seems to incorporate the same line of thinking of Bandura. This refers to the action of parents, which contributes greatly to the thought formation and structure of children’s personality. What ties these ideas is the fact that they learn by imitation and that imitation will be of those who are their biggest references: Father and Mother.^9,18^

In this sense, the findings found here corroborate the results of the study by Menezes et al. (2009), demonstrating the interaction of the behavior learned by these children with the increase in blood flow in given brain areas. If parents show children ways of using the mind, they possibly favor the increase in cerebral irrigation in doing so, enabling greater cognitive performance particularly related to the aspects of attention capacity. On the other hand, if parents do not stimulate this, in the case of this work, the computers are used by them and their children only in activities that require little functioning of the cognitive system, so there will probably be no increase in level of attention.^10^

The results of this study, however, with regard to the Control 3 group, raise questions about ratification possibly in relation to blood flow displacement only in focus and mental manipulation, which showed no statistically significant difference, yet in sustained attention and resistance to interference, found to be reduced compared to IDWC. These results highlight the performance increase of IDWC, also suggesting that the condition of being deaf increases visual attention as a compensation effect.

The present results still contradict the findings of Guerra-Ribas et al. (2010), which somehow lend credence to the previous line of thought, since Guerra-Ribas et al. (2010). These authors evaluated the stress and attention level in food handlers at a Public Hospital in Recife and found a reduction in the attention level in food handlers with more than 5 (five) years in the profession compared to handlers with less than 5 (five) years.^7^ In this context, they are professionals of manual labor, who make little use of the cognitive system compared to professionals that use computers and read.

These results may prove of great relevance to Human Resources professionals, in raising the importance of stigmatized deaf individuals, and may have an impact on shaping the work market. Therefore, further investigations are necessary, particularly because this work was of an exploratory nature and used a model with a small number of subjects.

Concluding, this study highlights the relevance sensory systems to cognitive processing.
